# Application programming interfaces for knowledge transfer and generation in the life sciences and healthcare

**DOI:** 10.1038/s41746-020-0235-5

**Published:** 2020-02-28

**Authors:** Stephen K Woody, David Burdick, Hilmar Lapp, Erich S. Huang

**Affiliations:** 1 Duke University School of Medicine, 701W. Main St, Durham NC, 27701 USA; 2 Stratus Medicine, 920S. Holgate St, Suite 104, Seattle, WA 98134 USA; 3 Center for Genomic and Computational Biology, Duke University, 101 Science Drive, Durham, NC 27708 USA; 4 Duke Health, Duke Forge and Duke Crucible, Suite 401, Davison Building, 100 Trent Drive, Durham, NC 27708 USA

**Keywords:** Translational research, Public health

## Abstract

Storing very large amounts of data and delivering them to researchers in an efficient, verifiable, and compliant manner, is one of the major challenges faced by health care providers and researchers in the life sciences. The electronic health record (EHR) at a hospital or clinic currently functions as a silo, and although EHRs contain rich and abundant information that could be used to understand, improve, and learn from care as part learning health system access to these data is difficult, and the technical, legal, ethical, and social barriers are significant. If we create a microservice ecosystem where data can be accessed through APIs, these challenges become easier to overcome: a service-driven design decouples data from clients. This decoupling provides flexibility: different users can write in their preferred language and use different clients depending on their needs. APIs can be written for iOS apps, web apps, or an R library, and this flexibility highlights the potential ecosystem-building power of APIs. In this article, we use two case studies to illustrate what it means to participate in and contribute to interconnected ecosystems that powers APIs in a healthcare systems.

## Introduction

In the late 19th and early 20th century as households became electrified for light, the concept of “appliances”—devices that might use electricity for other purposes, such as sewing machines and chafing dishes—was novel. A tally by the Southern California Edison Company reported that in 1904 there were 500 appliances in all of Southern California.^1^ Surprisingly, “the appliance plug is conspicuously absent” from Thomas Edison’s myriad patents.^1^ For decades, the principle means for connecting electrical devices was screwing them into the same Edison-type sockets used by light bulbs. This came with attendant inconveniences and hazards: for instance, connecting one’s flat iron meant that one would have to remove light bulb to do so, the power cord would become vexingly twisted when connecting it, and if the iron were dropped there was not an easily separable connection between the appliance and its power supply with the possibility of short circuit or shock. It was not until 1917–1937, years after Edison’s patent for the light bulb—that six manufacturers agreed on a standard receptacle for separable attachment plugs.^1^

In healthcare and life sciences, we are currently living in an era analogous to the early days of electricity; an uncomfortable interregnum between the clear promise of health data appliances/applications for improving health and the difficult reality of our data is equivalent to one-off custom hard-wiring or twisted cords dangling from Edison screw-type sockets. Health data are not readily transmittable across diverse sources, systems, and countries, and are typically housed in monolithic servers cordoned by technical, legal, ethical, and social roadblocks.^2^ While we are used to the myriad consumer “apps” that “plug-in” to the iOS or Android ecosystems, there is no equivalent in health care and the life sciences. Even when access to particular data is granted, making those data readily usable for the secondary purposes of generating and transferring knowledge is challenging. A widely held rule of thumb is that 80% of the time spent in creating an analytic data set is allocated to cleaning, linking, and merging data, while only 20% of the effort is applied to analyzing the data for insights or applying machine learning.^3,4^

**Fig. 1:**
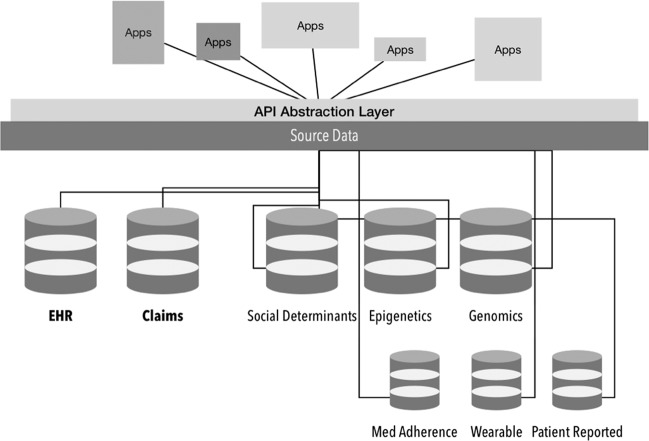
APIs and an abstraction layer on top of source data. An application programming interface (API) abstraction layer on top of data sources can help make data accessible through applications to facilitate the transaction of data via APIs.

The idea of a data warehouse for health data, developed decades ago for periodic reporting, should be reevaluated as applications—programs that make those data usable or actionable on a routine basis. “Secondary” data transactions tied to analytic workflows or triggering actions are becoming as important as “primary” use. And we must think of data less as a “stash” to be accessed only periodically, but as a flow of information that is constantly being used—analogous to how we think of electricity being ubiquitously available for everyday tasks. Therefore, it becomes more important to think of a layer on top of data sources that make it more easily accessed by applications via “application programming interfaces (APIs)” to facilitate the transaction of data to applications (see Fig. 1). These ‘interfaces’ represent the data equivalent of the ubiquitous 110 v wall socket, and applications become the equivalent of physical appliances.

A recent elegant example of this concept embraces the HL7’s Fast Health Care Interoperability Resources (FHIR) standard^5^ (which essentially provides a standardized container for data) to represent a patient’s entire EHR in temporal order (with all the idiosyncratic code in the EHR intact), and applied deep learning techniques to predict clinical outcomes (death, length of stay, and diagnoses) in a way that out-performed other clinically used predictive models.^6^

Recently, the Office of the National Coordinator for Health Information Technology (ONC) and the Centers for Medicare and Medicaid (CMS) announced a Proposed Rule intended to advance interoperability and support the access, exchange, and use of electronic health information.^7^ The Proposed Rule calls on health IT developers to use APIs and mandates that information about the technology be “exchanged, accessed, and used without special effort.”^7^

In this article, we illustrate what it means to participate in and contribute to interconnected ecosystems powered by APIs. We present two case studies of how we used APIs in an academic health system, and finally, present a way forward for developing and nurturing this ecosystem. The ONC proposed rule represents an important start; future practices and policies will need to advance the use of APIs for clinical data exchange from the electronic health record and other relevant data sources, not only for patient and clinician use, but also for other health care providers, payers, and for research.

Healthcare systems leaders who make decisions about IT teams and the IT teams themselves should rethink data infrastructure in healthcare so that modern applications and analytics can be delivered through APIs, services, and cloud-like environments. This will support the goal of facilitating knowledge generation while minimizing the burdens of navigating access to monolithic data silos.

### A brief overview of APIs

APIs are analogous to standardized electrical sockets, but instead of electricity, APIs perform the service of moving data. APIs are software ports that provide secure routes for moving data around a network or the internet; they are a mechanism by which an external program invokes another program’s function or methods. Accordingly, moving to an IT strategy that supports APIs is important because it standardizes the “socket” for sharing and using data.

#### Case Study #1: data provenance solution at Duke using APIs and microservices

In response to an incident of research misconduct at Duke University where a researcher altered data sets,^8^ our research team conducted a project called the “Duke Data Service” to capture data provenance from the time of data creation through to publication. Based on two of the authors’ early experience in developing a system for tracking provenance at Sage Bionetworks (where they have been working on the problem), we embarked on creating a solution using APIs and microservices. This was Duke’s first enterprise, service-based application, the first application designed and built from the ground up to implement a service-based architecture, and the first time we used cloud services as part of the deployment and execution process.

### Methods

At Duke University, much of the original data surfaces in core labs across campus (e.g., next gen sequencing). To limit the opportunity to corrupt data, we set out to capture and “fingerprint” data at the earliest possible point in the process. Our intent was to automate the process of taking data off of the equipment, create a hash, and store the data in a secure storage system. On the deployment side, we used the Heroku platform. Execution services include Ruby (Rails), RabbitMQ, PostgreSQL, Neo4j and Elasticsearch. Adhering to a service-based architecture meant that we would encapsulate all functions within services with clearly defined APIs. (We posted all documentation on the GitHub repository https://github.com/Duke-Translational-Bioinformatics/duke-data-service). Documenting a specification for each API is the genesis of each service. We used Blueprint (https://apiblueprint.org) to document our APIs and Dredd (https://dredd.readthedocs.io/en/latest/) to test them. We automated the process of testing and deployment. Today, the APIs go through over 5000 tests prior to deployment. Once passed, the process deploys the code. This process improves quality and facilitates quick deployment of fixes and new functions.

Duke Data Service breaks down into the following functional categories:

- Authorizations
- Projects
- Folders and files
- Storage (Swift object storage)
- Metadata
- Search
- Provenance
- Core facility workflow

The University IT group provides the authentication service for user and service accounts. The Data Service employs a project concept and provides folder structures and access management. In addition, the Data Service provides a flexible metadata layer allowing the user to store and query any desired metadata. OpenStack’s Swift object storage is our original storage implementation, however, we designed the Data Service to easily add new data storage options such as cloud object storage (Amazon’s S3), archive storage (Amazon’s Glacier), etc.

### Results

Our research community easily understands the base data services and file services grouped by project with some metadata. There is an easy-to-use mobile device and web-based interface allowing access to projects with folders and files. APIs are straightforward, documented with examples and easy to use. Using these basic storage services provides initial provenance. As data moves through various transitions and translations, researchers need a more sophisticated provenance capability. Based on the W3C standard for provenance (https://www.w3.org/2001/sw/wiki/PROV), we are adding new provenance services.

The result of our work is a service-based provenance storage called the Duke Data Service. The Duke Data Service provides, through a service-based API, access to significant storage (120 terabytes), and the ability to assign/move data from one custodian (for example, the core lab) to another (for example, a researcher). We recently added another storage provider, Dell/EMC’s Elastic Cloud Storage (1.8 petabytes) without affecting existing users and simply adding another storage option.

The Data Service team designed the services with the expectation that others outside of the Data Service team would eventually utilize Data Service’s capabilities. During the first seven months of 2019, the Data Service received on average 46,300 files per month totaling almost 7500 GB per month.

### Discussion

Because our solution used APIs and microservices, it enabled an ecosystem where others could build off our work. For example, the data-producing core labs at the Duke University Center for Genomics and Computational Biology (GCB), sought to better automate its genomics data management and delivery processes. GCB’s research IT group (note that this team is entirely separate from the team that built Duke Data Service), without our prompting, recognized that the Duke Data Service provides key enterprise-level infrastructure building blocks that enabled it to create an application on top of the Duke Data Service API that focuses on uploading, sharing, transferring, and downloading high-volume genomics data from a command-line shell environment. In essence, GCB has independently written a series of “appliances” or applications leveraging the Duke Data Service “sockets” or APIs. The applications were subsequently integrated into the core lab’s data generation and delivery pipeline. By connecting to the Duke Data Service for storage of laboratory output, it automatically starts a provenance chain for these data, one of our original motivating goals. The application created by the GCB team is written in the Python language, and thus uses a technology stack distinct from the Duke Data Service. Such loose coupling, and ability for client applications to use the technology stack most appropriate for them (Python is much more widely used in the computational genomics domain than, for example, Ruby), is among the key benefits enabled by a service-based architecture. In GCB’s latest release, they added a web-based user interface connecting into the same APIs making it even easier for the cores’ lab staff to manage data delivery, and for research labs to receive the data at the most useful location.

### Data provenance API source code

Because data provenance is important for all research institutes, we share the source code, the architecture and the methods by which we are attempting to solve the problem publicly on Github (https://github.com/Duke-Translational-Bioinformatics/duke-data-service).

#### Case Study #2: providing up-to-date Institutional Review Board (IRB) information through APIs—an useful abstraction layer

A more recent implementation of an API strategy demonstrates the value of an API as an abstraction layer. Internal audit cited a department for not keeping access privileges to private health information (PHI) in sync with the IRB’s official record of key personnel. To address this issue, we created two API endpoints that provide the status of the IRB approval (active or not active) and a list of current key personnel (those that may access PHI). Our prior IRB system and database did not provide any programmatic mechanism or direct insight to this information. Moreover, understanding the IRB approval status was not as simple as looking up a status. There were other statuses that, in combination with the expiration data, indicated that the IRB approval was active.

### Methods

Instead of replicating this business logic in multiple systems, we centralized it in the API. We were fortunate that a number of years ago we contracted with the IRB vendor to help us create an extract of the data into a reporting data mart, which was the source of data for the API. The reporting data mart is updated daily, which is adequate for our purposes. Systems that needed the active status of a protocol and the key personnel list can simply call the API. The API is REST-based and does not require the calling program to be written in any particular language. Most modern programming languages support REST calls.

Not only were we able to encapsulate the business logic, but during the implementation, we changed IRB vendors. The change involved creating a new reporting database and changes in logic determining if the IRB is active. We modified the API to access the new data mart and the business logic. No changes were necessary to any application using the API, and everything was completely transparent.

Implementation of this new API added an API manager or service bus, Kong. The API manager authenticates each call and performs message traffic regulation such as load balancing and throttling. The API manager provides greater insight into communication and message traffic.

On the user side, a few lines of code—without the need for a professional programmer—can be used to populate up-to-date IRB data even to database-style applications. In fact, most modern frameworks for API documentation now automatically provide code that can be readily used to access those APIs.^9^

### Discussion

In this example, we see two clear benefits of the API model: (1) keeping IRB business logic out of other applications by centralizing it in one place and (2) isolation of the IRB application allowing us to swap IRB vendors without impacting applications using the API.

As with many institutions like Duke, there are API construction activities in many departments. These activities tend to be disjointed, lack common standards, and have no mechanisms for preventing redundancy. The next two major challenges we plan to address in institutionalizing the API-based architecture are (1) organizing information and documentation on APIs in a common location so others can search for and use existing APIs (rather than creating new APIs every time) and (2) improving the maturity of APIs.

## Discussion

To recall our earlier analogy using the 110 Volt AC socket, providing standardized and well-documented “data sockets” via APIs provides many benefits in healthcare and the life sciences that are similar to the benefits that sockets have provided for electrical appliances in our daily life.

1. Standardized API interfaces simplify building applications: as we note in our examples, once a uniform and documented set of APIs was created it became very easy for developers from an entirely different team than the API developers to create fit-for-purpose applications such as an application for delivering large next generation sequencing data to customers from a genomic core laboratory or incorporating up-to-date IRB status and key personnel to a research database.
2. Applications become easier to maintain: since applications are built against APIs, if the system behind the API interface layer changes, there are minimal if any changes the developer must make. To use the AC socket analogy, if you add solar panels to your house or your power company switches to a different plant, your microwave oven continues to function without any intervention. As noted in our IRB example, even though the underlying IRB system changed entirely, the were no changes necessary for the consumer of the API.

APIs encourage ecosystems: from the time of Edison on, standardized methods for storing and delivering electricity has encouraged an ecosystem of devices that make that electricity useful in our daily lives, whether via appliances or now, automobiles. As data acquires the same ubiquity and utility as electricity, APIs represent a standard for making data useful through applications. Alphabet, the parent company of Google, provides public access to close to 200 different APIs, many of which will be familiar to the reader (at least through apps such as Google Maps or Google Assistant), such as those for street view, directions, and search. While Google itself uses these APIs for its own apps, other companies use them as well through their own applications, such as Uber or AirBnB.

Assuming one accepts the general principle of APIs, an important issue to be addressed is the lack of data standards. This is a fair question and addresses a fundamental “chicken or the egg” problem in health informatics. Do we await standards before exposing them via application programming interfaces? Or do we “prime the pump” and begin exposing data via interfaces and allow standards to develop with increasing ubiquity and use of data? HL7’s FHIR standard is an important first step for health-related data and is widely embraced as a proposed standard even while its actual use is still very limited. In many ways, FHIR embraces many best practices learned from the widespread adoption of APIs outside of healthcare. One of the most important of these principles is providing human and machine-readable documentation of an API. Whether an API exposes data as XML (Extensible Markup Language), JSON (Javascript Object Notation), YAML (YAML Ain’t Markup Language), or Protobuf (Protocol Buffers), if it is readily parse-able, API documentation can make it relatively easy for application developers to consume those data. In fact, many modern frameworks for building APIs automatically generate documentation, and further, generate programmatic libraries such that programmers merely need to install a pre-built library to use an API. Therefore, all the tools to make an API immediately useable by someone else can be seamlessly produced as artifacts of building an API.

This begs an additional consideration: the Google Maps API, as well-documented and understood as it may be, is not useable by a layperson; software engineers must write an application—whether it is the Google Maps app or the AirBnB app on one’s phone—to make it useful to non-programmers. Application programming interfaces are necessary, but not sufficient, for making data readily useable. Just as electricity requires engineers and manufacturers with special capabilities to produce refrigerators or plug-in hybrids, producers of health data APIs require counterparts with the skills to write health data applications for use cases such as AI-driven clinical decision support or clinical trial matching. In healthcare and the life sciences, creating this new category of application developers requires recognition of this need and a cultural evolution that embraces a new professional category of personnel who have both domain expertise in the health and life sciences and software engineering skills.

Some sectors in life sciences are building data ecosystems, for example, in 2015, the National Institutes for Health Big Data to Knowledge (NIH BD2K) Center for Big Data in Translational Genomics (CBDTG) pioneered the development of shared APIs to connect genomics repositories.^2^ Genomic data sets suffered from siloed systems that lacked common standards across diverse and geographically disparate sites, and there were clear incentives for facilitating access to the data so they could be compared.

## Conclusion

Our colleague, Amy Abernethy, notes in an AMIA keynote that “the more that we use data, the clearer the river of data gets”.^10^ We suggest that APIs for life science and health data provide the wellspring for such a river. At the same time, APIs by themselves are not enough. Developers who are skilled at both creating and consuming APIs and are conversant in the health and life sciences are necessary too. As with any new technology, new job categories are essential to making them useful to our communities. Standards are necessary too, and the question arises whether standards need to occur first or whether increasing data liquidity will provide opportunity for the community to develop standards informed by real-world usage. If this is the case, we will also need to learn from our peers in the technology sector the best practices for API design and documentation that facilitate active use and a crowdsourcing of effective standards. FHIR represents a promising start in this direction.

Undoubtedly, significant culture change is required to build toward a vision of API-driven data interchange. Again, there is much to learn from the technology sector in observing the pace of innovation that has accompanied widespread adoption of APIs. Companies like Stripe or Twilio provide myriad customers capabilities for their apps via the Stripe payment or Twilio messaging APIs. Traditional industries such as automobile manufacturing or banking are responding to this by rapidly expanding their cohorts of API and application developers; it is clear that applications are the “appliances” of our time. Our experience in an academic health system is that API development is a means to begin developing an ecosystem and culture that focuses on the usage as opposed to the warehousing of data. No doubt, as with any culture change, it will take time to live up to the promise of APIs and applications in health, but the historical parallels of electrification and the tech sector’s API economy provide reassurance that the investment will be rewarded.

## Data Availability

For Duke Data Service, we posted all code and documentation on the GitHub repository https://github.com/Duke-Translational-Bioinformatics/duke-data-service.
