# Publisher Correction: Application programming interfaces for knowledge transfer and generation in the life sciences and healthcare

**DOI:** 10.1038/s41746-020-0271-1

**Published:** 2020-04-09

**Authors:** Stephen K. Woody, David Burdick, Hilmar Lapp, Erich S. Huang

**Affiliations:** 1 Duke University School of Medicine, 701W. Main St, Durham, NC 27701 USA; 2 Stratus Medicine, 920S. Holgate St, Suite 104, Seattle, WA 98134 USA; 3 Center for Genomic and Computational Biology, Duke University, 101 Science Drive, Durham, NC 27708 USA; 4 Duke Health, Duke Forge and Duke Crucible, Suite 401, Davison Building, 100 Trent Drive, Durham, NC 27708 USA

**Keywords:** Translational research, Public health

Correction to: *npj Digital Medicine* 10.1038/s41746-020-0235-5, published online 28 February 2020

The original version of the published Article contained an error in the last sentence of the first paragraph of the Introduction. To improve clarity “It was not until 1917–1937, years after Edison’s patent for the light bulb…” has been changed to “It was not until 1917, 37 years after Edison’s patent for the light bulb…”. The HTML and PDF versions of the Article have been corrected.

